# Scleredema Adultorum of Buschke Associated With Diffuse Eosinophilia and Hepatitis B Virus Infection

**DOI:** 10.7759/cureus.38300

**Published:** 2023-04-29

**Authors:** Divya R Patel, Neel Vora, Rajvi Chaudhary, Vishnu Sharma, Nikunj V Dadhaniya

**Affiliations:** 1 Internal Medicine, B.J. Medical College, Ahmedabad, IND; 2 Rheumatology, Apollo Hospitals, Ahmedabad, IND

**Keywords:** vein grooves, eosinophilia, scleroderma mimic, hepatitis b virus, scleredema adultorum of buschke

## Abstract

Scleredema adultorum of Buschke is a rare condition that presents as a scleroderma mimic and portends a diagnostic challenge to the clinician. It may be associated with monoclonal gammopathy, upper respiratory tract infection, or type II diabetes mellitus. In addition, it is associated with dermal collagen and aminoglycan deposits that cause the skin to thicken and stiffen. Typically, thickening and tightening begin in the neck and progress to the upper body, including the face, scalp, shoulders, and trunk, but sparing the palms and soles. Patients with minor skin involvement may not suffer any symptoms, whereas those with significant skin disease may develop stiffness and functional impairment. There are rare reports linking scleredema adultorum of Buschke with several infections such as human immunodeficiency virus infection, acquired immunodeficiency syndrome-related lipodystrophy syndrome, and streptococcal infection of the upper respiratory tract. Here, we present a case of scleredema adultorum of Buschke associated with hepatitis B infection.

## Introduction

Scleredema adultorum of Buschke is a skin condition characterized by the increased build-up of collagen and aminoglycans in the dermis. These plaques are non-pitting, woody lesions. When pinched, the epidermis covering the affected area becomes wrinkled or develops a peau d’orange appearance. Typically, lesions are limited to the upper back, shoulder, and nape of the neck. Involvement of the face and tongue might also lead to difficulty in opening the mouth or eyes, dysarthria, or mastication issues. Hands and feet are not affected.

There are three varieties of scleredema adultorum of Buschke, namely, type 1, the traditional kind, which develops following respiratory infections; type 2, linked to hematological disorders, most frequently paraproteinemia; and type 3, associated with type II diabetes mellitus.

Here, we present the case of a male patient in his mid-20s who complained of skin tightening involving the back of his neck and proximal upper and lower limbs. He was later diagnosed with scleredema adultorum of Buschke associated with hepatitis B viral infection.

## Case presentation

A male patient of South-Asian ethnicity from India in his mid-20s presented to our hospital with skin thickening involving the neck, upper back, arm, forearm, and thighs with itching for three months. The patient was relatively asymptomatic before three months when he started developing skin tightening and stiffness, which initially involved the upper back and neck region but gradually spread to the arms, forearms, and thighs. He denied any history of Raynaud’s phenomenon. The patient denied fever, joint pain, oral ulcers, abdominal pain, breathlessness, hematuria, bruising, dizziness, chest pain, hemoptysis, visual blurring, diplopia, or hematochezia.

History was otherwise unremarkable for tuberculosis, hypertension, thyroid disorders, psychiatric diseases, or autoimmune diseases. He did not have any significant family history of autoimmune or chronic illnesses. The patient denied using tobacco products, alcohol consumption, or illicit drugs.

**Figure 1:**
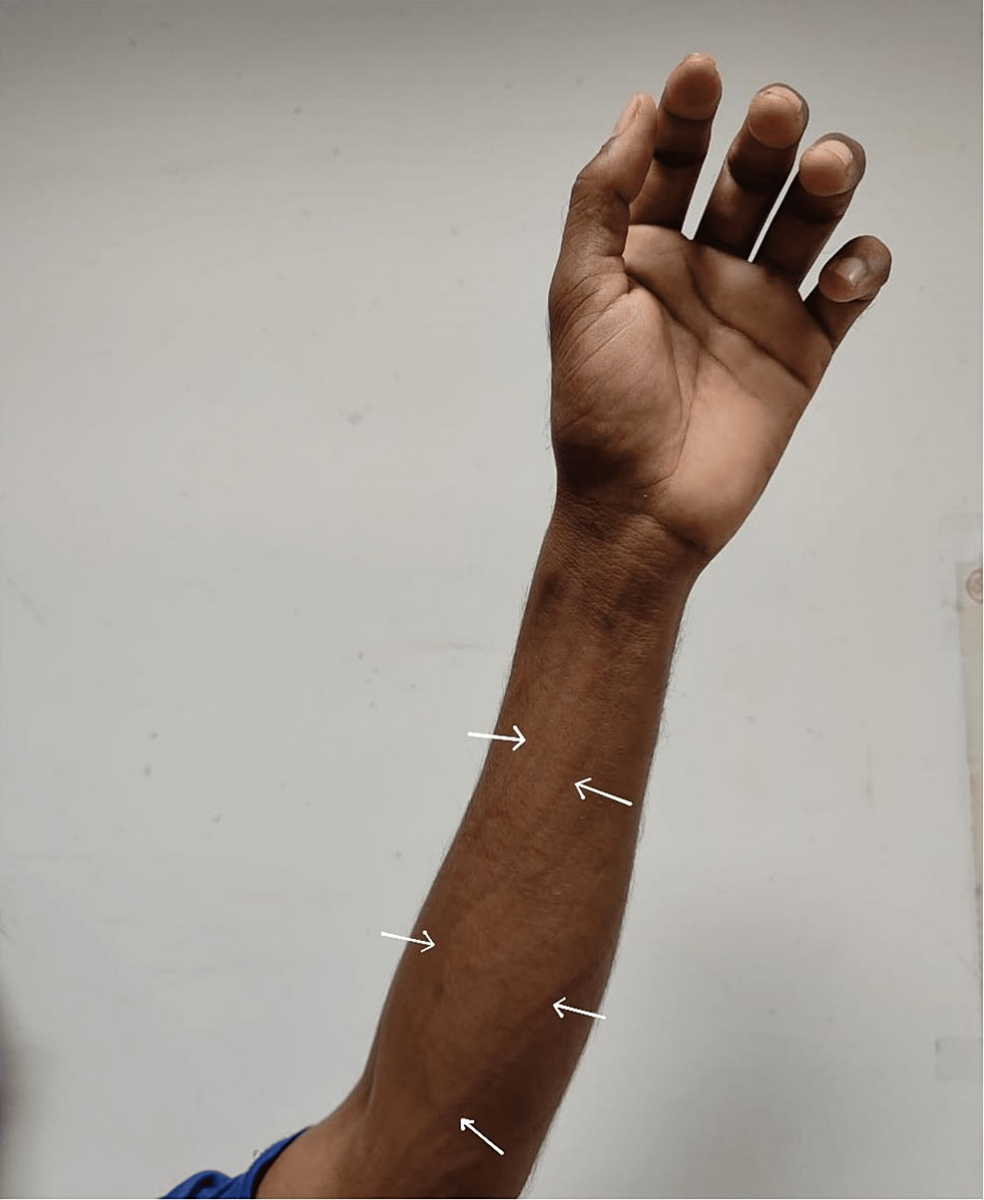
Vein grooves on the left arm. White arrows show vein grooves on the left arm.

The patient was oriented to time, place, and person. Physical examination revealed extensive skin sclerosis that appeared hyperpigmented, along with asymmetrical skin induration over the back of the neck, back, abdomen, and bilateral upper and lower limbs, but sparing the palms and soles. Overhead elevation of the upper limbs showed prominent venous grooves (Figure 1). His vitals were unremarkable, as was the remainder of the physical examination.

His routine investigations showed eosinophilia (907 cells/mm^3^, 13% in a total of 6,980 white blood cells), positive hepatitis B surface antigen (HBsAg), and elevated alanine transaminase and alkaline phosphatase (Table 1). The patient tested negative for hepatitis C and hepatitis A virus infection.

**Table 1:** Laboratory investigations.

Investigations	Result	Biological reference range
Hemoglobin	10.80 g/dL	13–17 g/dl
Red blood cells	4.44 × 10^6^/mm^3^	4.5–6.5 × 10^6^/mm^3^
White blood cells	6.98 × 10^3^/mm^3^	4–10 × 10^3^/mm^3^
Hematocrit	34.10%	40–50%
Polymorphs	58.00%	40–80%
Lymphocytes	22.00%	20–40%
Eosinophils	13.00%	1–6%
Monocytes	7.00%	2–10%
Basophils	0.00%	0–1%
Absolute neutrophil count	4,048 cells/mm^3^	1,500–7,000 cells/mm^3^
Absolute lymphocyte count	1,536 cells/mm^3^	1,000–4,800 cells/mm^3^
Absolute eosinophil count	907 cells/mm^3^	50–500 cells/mm^3^
Absolute monocyte count	489 cells/mm^3^	300–900 cells/mm^3^
Absolute basophil count	0 cells/mm^3^	
Platelets	305 × 10^3^/mm^3^	150–500 × 10^3^/mm^3^
Hepatitis B surface antigen	Reactive	
Serum alanine transaminase	123.0 IU/L	0–45 IU/L
Serum alkaline phosphatase	355.90 U/L	41–137 U/L

**Figure 2:**
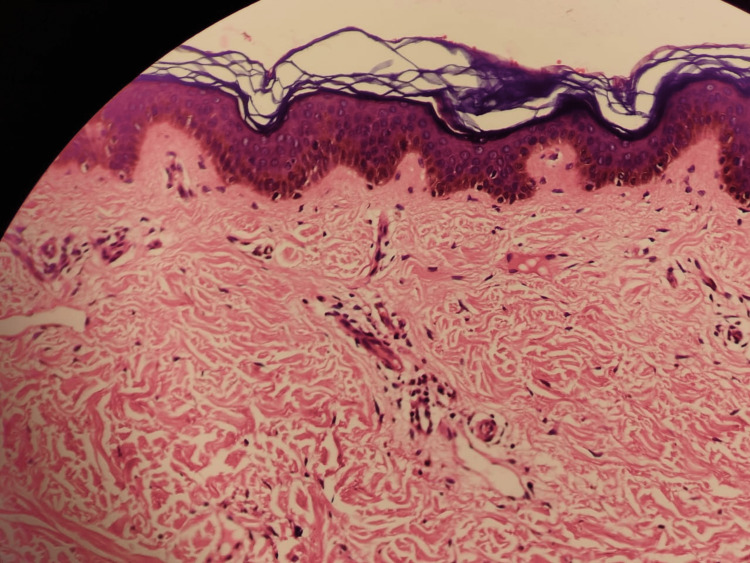
Histopathological image showing the normal epidermis with mild hyperkeratosis. Hyperkeratosis is the thickening of the outer layer of the skin.

**Figure 3:**
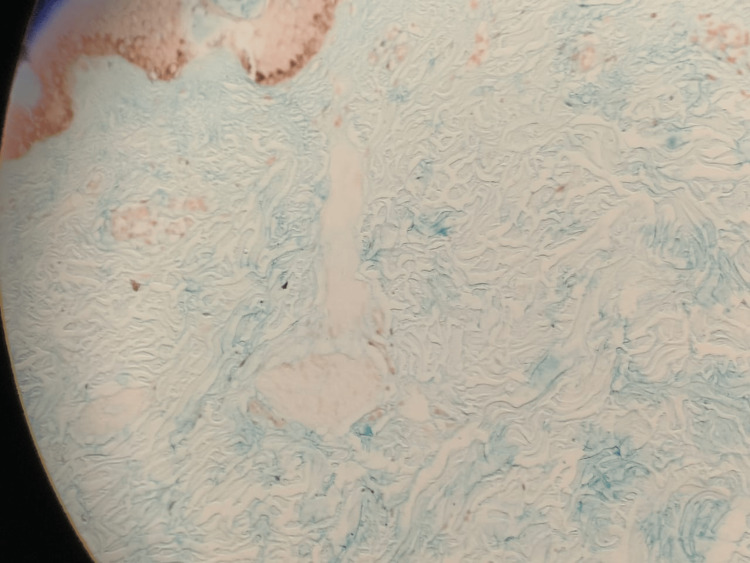
Histopathological image with Alcian blue stain. Alcian blue stain is used to stain the acidic mucus and acetic mucins.

Ultrasound of the abdomen and pelvis showed a normal-appearing liver and no evidence of cirrhosis or fibrosis. A punch biopsy of the lesions from the dorsal aspect of the left forearm was consistent with the diagnosis of scleredema adultorum of Buschke. Histopathology of the skin section showed a normal epidermis with mild hyperkeratosis (Figure 2). The superficial and deeper dermis showed increased collagenization, proliferation in capillaries with endothelial thickening, chronic perivascular inflammation, and mucin-like material between the collagen fibers. The deep dermis showed an upward position of eccrine glands with mild atrophy. Thick vascularized collagen fibers separated the subcutaneous fat, which had moderate interstitial lymphoplasmacytic infiltration and the presence of mucin. The Alcian blue stain was positive for aminoglycans (Figure 3). Overall, the histology was suggestive of a clinical diagnosis of scleredema adultorum of Buschke. However, scleredema did not explain the raised eosinophil count. Overall, the findings suggested the diagnosis of scleredema adultorum of Buschke.

Oral prednisolone at a dose of 1 mg/kg/day was administered. He was discharged with this medication and was asked to follow up after a month.

## Discussion

Several sclerotic disorders such as systemic sclerosis, scleromyxedema, eosinophilic fasciitis, and nephrogenic systemic fibrosis can share clinical features with scleredema. To differentiate between these conditions, a comprehensive clinical examination, a review of the histopathological findings, as well as some laboratory studies are helpful. Our patient initially complained of bilateral lower limb heaviness and stiffness for three months, bilateral upper limb heaviness and abdominal muscle stiffness for two months, a headache for 10 days, and diffuse skin tightening with infrequent itching. An initial investigation revealed an elevated eosinophil count on a complete blood count test. This finding, along with the typical sclerotic features and groove sign, pointed toward a diagnosis of eosinophilic fasciitis. However, the punch biopsy report of our patient was more conclusive of scleredema adultorum of Buschke rather than eosinophilic fasciitis, whose typical biopsy findings include deep fascia with edema and lower subcutis infiltrated with lymphocytes, plasma cells, histiocytes, and eosinophils. The absence of heart, lung, and kidney involvement, and a usual antinuclear nuclear antibody profile, also ruled out systemic sclerosis.

The exact prevalence of scleredema is not known. Although scleredema is typically a rare condition, a prospective study of 484 people with diabetes in California reported a 2.5% prevalence of scleredema, suggesting that scleredema is underrecognized [1]. All racial and ethnic groups are affected by scleredema, and there is no obvious sex difference in the propensity of the disease [2]. Scleredema can affect anyone, including children and adults [3,4].

The pathogenesis of scleredema is not well understood. Unnaturally high levels of collagen and mucin may accumulate in scleredema caused by diabetes due to irreversible glycosylation of collagen and altered collagenase activity [5]. Additionally, hypoxia and microvascular injury may have profibrotic consequences [6]. Increased collagen production may be a direct result of streptococcal hypersensitivity or paraproteinemia in cases of scleredema unrelated to diabetes.

The etiology of scleredema etiology determines how it manifests clinically and how it progresses. Scleredema associated with diabetes mellitus. The development of diabetes mellitus-associated scleredema is typically gradual and slow-progressing. Typically, the extremities remain unaffected. However, the posterior neck, upper back, and chest are frequently affected areas [2]. It is usually a chronic condition.

Scleredema that arises after an infection presents a more abrupt onset. Typically, scleredema symptoms start a few weeks after the initial infection [7,8]. The cervicofacial region is primarily affected. In more severe cases, the mouth and pharynx may be involved, which can cause dysphonia and dysphagia. After a few months, post-streptococcal scleredema frequently experiences total clinical regression [9]. Infection-related scleredema is much more prevalent in children and young adults.

Scleredema related to monoclonal gammopathy shares several clinical characteristics with scleredema associated with diabetes mellitus. The slow onset and slow course are its hallmarks [10,11].

In most patients, symptoms and signs of scleredema are restricted to the skin. Systemic involvement is rare and is seen only in severe forms of the disease. Ocular (ocular muscle palsy), gastrointestinal (dysphagia), respiratory (dysphonia), muscular (myositis), and cardiac symptoms have been reported in the literature [2,3,12-14]. Death has occurred infrequently as a result of systemic involvement [14,15].

Key histopathologic findings in scleredema include [16] normal epidermis, thickened reticular dermis, and pronounced swelling of collagen bundles that appear to be spaced apart by varying amounts of mucin. Slight perivascular and periadnexal lymphocytic infiltrates also may be present. Because the condition is benign and often asymptomatic, treatment of scleredema is not always necessary. There is no best approach to treat scleredema, but various modalities such as phototherapy, immunosuppressants, radiation therapy, and physical therapy have been documented in the literature. No single treatment appears to be uniformly effective [17].

## Conclusions

Scleredema adultorum of Buschke is a rare condition that presents as a scleroderma mimic and portends a diagnostic challenge. There is no best approach to treat scleredema. This case presentation is significant because scleredema adultorum of Buschke is a close mimicker of scleroderma. We have a new observation that this patient’s scleredema adultorum of Buschke was associated with hepatitis B virus infection, which is a new association. Therefore, clinicians must look for other infectious etiologies than the ones already mentioned in the literature.
